# *APOE* Gene Variation’s Impact on Cardiovascular Health: A Case-Control Study

**DOI:** 10.3390/biomedicines12030695

**Published:** 2024-03-21

**Authors:** Aya Badeea Ismail, Özlem Balcıoğlu, Barçın Özcem, Mahmut Çerkez Ergoren

**Affiliations:** 1 Department of Medical Genetics, Faculty of Medicine, Near East University, Nicosia 99138, Cyprus; 2 Department of Cardiovascular Surgery, Faculty of Medicine, Near East University, Nicosia 99138, Cyprus; drbalcioglu@hotmail.com (Ö.B.); barcin.ozcem@neu.edu.tr (B.Ö.)

**Keywords:** cardiovascular diseases, *APOE* gene, polymorphism, rs7412, rs429358

## Abstract

Chronic venous insufficiency (CVI) is a common medical condition characterized by impaired functioning of the venous system in the lower extremities. It leads to various symptoms, including varicose veins, leg edema, and skin pigmentation. It is believed that a combination of genetic and environmental factors affect the development of CVI. The *APOE* gene is of particular interest in this context, as it plays a role in lipid metabolism and inflammation. The ε4 allele (rs429358) has been associated with an increased risk of Alzheimer’s disease, while the ε2 allele (rs7412) has shown a protective effect against Alzheimer’s disease but a strong association with cardiovascular inflammation. This research aimed to investigate the presence of *APOE* gene variants in individuals with chronic venous insufficiency disease and validate the relationship between this gene and cardiovascular diseases. The study analyzed the expression of *APOE* gene variants in varicose vein tissue samples from patients and a normal vein in the control group. The results indicated no significant expression of the ε4 allele in either group. However, there was a significant decrease in the expression of the ε2 allele in the patient group. Additionally, a negative correlation was observed between the two single nucleotide polymorphisms (SNPs) in vein tissue. The lower expression of the ε2 allele in patients suggests a potentially reduced risk of cardiovascular disease in these individuals. Consequently, there appears to be a weaker association between the expression of the APOE gene ε2 allele and cardiovascular diseases.

## 1. Introduction

Chronic venous insufficiency (CVI) is a medical condition affecting the venous system. The pathophysiology of CVI involves a complex interplay of venous valve dysfunction and venous hypertension, spider veins, asymptomatic varicosities, large painful varicose veins, edema, hyperpigmentation, and lipodermatosclerosis of the skin, and ulceration [1]. Age, female gender, diabetes, obesity, high blood pressure, smoking, and cardiovascular disease were identified as independent predictors of CVI. Age and being female were specifically associated with a higher risk of varicose veins, telangiectasia, or reticular veins [2]. The diagnosis of chronic venous disease relies on the CEAP (Clinical, Etiological, Anatomical, and Pathophysiological) classification system. The annual incidence of CVI is about 150,000 and has a 3–1 female predominance [3]. The prevalence of chronic venous insufficiency (CVI) ranges from less than 1% to 40% in women and less than 1% to 17% in men [4]. However, beyond the age of 55, the condition becomes more prevalent in men [5].

The evident and sustained rise in cardiovascular disease rates in the past few years can potentially be attributed to the industrial growth of the global economy. This shift from physically demanding occupations to sedentary jobs associated with technology-driven culture, longer working hours, and extended commutes has led to reduced leisure time for recreational activities. As such, studies have indicated a correlation between inactivity, dietary patterns, smoking behaviors, and obesity, which play substantial roles in cardiovascular pathophysiology [6].

Previous research has indicated that the pathophysiology of venous and arterial vascular diseases shares similarities and is interconnected [7,8]. However, there is a lack of research specifically examining the relationship between venous disease and cardiovascular disease [9]. In an effort to address this gap, Prochaska et al. (2021) conducted an epidemiological study as part of the Gutenberg Heart Study to explore the association between cardiovascular disease and chronic venous insufficiency (CVI). Their findings demonstrated that cardiovascular disease and CVI are interdependent and share common risk factors [2].

In recent decades, the field of human genetics has increasingly acknowledged the role of specific genes in the potential development of cardiovascular diseases [10,11].

Apolipoprotein E (APOE) is widely recognized as a significant gene implicated in the pathophysiology of cardiovascular diseases, neuropsychiatric diseases, diabetes, and nephropathies [12]. The *APOE* gene resides in the chromosomal region 19q13.2, Exon 4 of the gene contains two frequently occurring single nucleotide polymorphisms (SNPs): rs429358 (388 T>C) representing the ε4 allele and rs7412 (526 C>T) representing the ε2 allele. Additionally, the combination of the 388 T and 526 C alleles represents the ε3 allele (wild-type) that is frequently seen in populations. Generally, the alleles ε2 and ε4 are regarded as pathogenic [13]. Allele frequencies for rs429358 and rs7412 are 0.1425 and 0.06542, respectively, in the entire gnomAD population, which includes sequencing data from 100,000 subjects from various disease-specific and population-specific genetic studies [14].

Epidemiological studies show that the common variants of the *APOE* gene have an effect on health and cardiovascular risk, mostly through small changes in the lipid profile. The ε4 allele represented by the rs429358 polymorphism is widely recognized as a prevalent genetic risk factor for the development of Alzheimer’s disease (AD). On the contrary, the less prevalent ε2 allele, associated with the rs7412 polymorphism, tends to exhibit a protective effect against Alzheimer’s disease but is notably linked to an increased risk of cardiovascular diseases (CVDs). Lastly, the ε3 allele is generally considered to have a neutral impact on Alzheimer’s disease (AD), lipid profiles, and cardiovascular health [15]. This complicated interaction of APOE alleles emphasizes the multiple functions that these genetic variations play in health and illness. Insights gleaned from research on mice with abnormal *APOE* genes reveal the profound impact of APOE expression on lipoprotein profiles. The absence of APOE expression is implicated in the modification of lipoprotein profiles, contributing to the manifestation of a spectrum of disorders ranging from cardiovascular diseases and neurological disorders to type II diabetes, compromised immune responses, and even a reduction in lifespan [16]. Delving into specific genotypic variations, individuals with the homozygous ε2 genotype have a two-fold higher risk of developing disorders related to the obstruction or rupture of peripheral vasculature [17]. These findings emphasize the significance of comprehending the precise genetic makeup of the *APOE* gene and its consequences for cardiovascular health.

The presence of APOE has been observed to have diverse effects on vascular function, including its role in preserving the integrity of the blood–brain barrier, modulating inflammatory responses, and platelet aggregation [18]. The APOE ε4 isoform has been found to be correlated with elevated carotid intima-media thickness as well as higher levels of LDL cholesterol and APOB concentrations [19]. The APOE protein is found in various cell lines in the brain, spleen, kidneys, sex glands, adrenal glands, macrophages, and vascular smooth muscle cells. However, its primary expression is most commonly observed in the liver; it plays a vital role in important physiological processes, including the metabolism of lipoproteins, modulation of fat-soluble vitamins, regulation of glucose and energy, signal transduction pathways, metastasis, and angiogenesis [20,21].

Building on this foundation, the overarching objective of the current study is to investigate the presence of *APOE* gene variations among individuals with venous insufficiency disease and to validate the potential association between these variations and cardiovascular diseases.

By exploring this relationship, the study aims to enhance our understanding of the genetic factors involved in both venous disease and cardiovascular conditions.

## 2. Materials and Methods

### 2.1. Vascular Material Collection and Listing

A total of 115 great saphenous vein samples were collected from the remains of varicose and bypass operations at Near East University Hospital (NEUH). The collected material was properly labeled and quickly frozen to preserve it until the collection phase was completed. These samples were divided into two groups: the patient group (62 samples) and the control group (53 samples). The experimental study, approved by the Near East University Scientific Review Board (Approval ID: YDU/2020/79-1034. Approval date 28 May 2020), strictly followed ethical standards. This research was carried out at the Near East University DESAM Institute Molecular Medicine Laboratory. All procedures in the study adhered to the high ethical standards set by the institutional research committee and the ethical principles outlined in the 1964 Helsinki Declaration and its subsequent amendments. Informed consent was obtained from all participants, ensuring transparency and respecting each participant’s autonomy.

### 2.2. Primer Optimization, Gradient PCR and Gel Electrophoresis

To prepare the primers, the 100 µM primer stock was diluted to create working primers at a concentration of 10 µM. This was done by combining 10 µL of the primer with 90 µL of dH_2_O. To determine the optimum temperature conditions for quantitative real-time PCR (qPCR), a gradient PCR was performed using the Applied Biosystems Veriti 96-well thermal cycler (Applied Biosystems™, Göteborg, Sweden). The following parameters were used: initial denaturation at 95 °C for 2 min, 40 cycles of denaturation at 95 °C for 30 s, annealing at 58–63 °C for 30 s, and extension at 72 °C for 45 s, followed by a final extension at 72 °C for 7 min. All the reactions for both gradient and qRT-PCR were carried out in a category II laminar flow hood to limit the risk of contamination; furthermore, all the reagents, plasticware, and pipettes were sterilized and designated for PCR. Wild-type APOE DNA served as the template, expecting a band size of 130 bp for rs429358 and 216 bp for rs7412.

A 3% concentrated gel made with Sigma agarose (Merck KgaA, Darmstadt, Germany) was used to electrophorese the PCR products. Six grams of agarose were mixed with 250 mL of TAE buffer. The mixture was heated until it became clear. Then, it was cooled down for 1–2 min before being poured into a 20 cm × 20 cm tray. 4 µL of ethidium bromide was added to the mixture and mixed very well. The gel mixture was poured into the tray and left to solidify. Next, 8 µL of each PCR product was mixed with 2 µL of loading dye (Thermo Scientific, Pittsburg, PA, USA) and then loaded into the well. Lastly, 3 µL of 50 Base Pair DNA Ladder was loaded alongside the samples. The samples were run at 120 volts by a Bio-Rad electrophoresis device (Bio-Rad, Hemel Hempstead, UK). The process took about 1 h and 30 min. To eliminate non-specific bands, MgCl_2_ and glycerol were employed. Visualization of the bands was achieved through an ultraviolet transilluminator (DNR Bio Imaging System, Jerusalem, Israel).

### 2.3. RNA Isolation, cDNA Synthesis, and Gene Expression Analysis 

The process of isolating total RNA from tissue was performed using a Hibrigen Hibrizol ready-to-use reagent for total RNA isolation from tissues and cells (Hibrigen Biotechnology Ltd., Gebze, Turkey). Tissue samples were mechanically crushed and homogenized in 1 mL of Hibrizol reagent solution, followed by the addition of 0.2 mL of Chloroform; the mixture was vortexed and incubated for 2–3 min at room temperature. The samples were then centrifuged at 2 to 8 °C at 12,000× *g* for 15 min. Centrifugation separated the mixture into three distinct phases: a lower phenol-chloroform phase containing protein and lipids for isolation, an intermediate phase containing DNA, and an upper aqueous phase containing RNA. The upper phase, containing RNA, was transferred to new tubes. Then, it was further mixed with 0.5 mL of isopropyl alcohol and incubated for 10 minutes at 15–30 °C. After centrifugation at 2 to 4 °C (12,000× *g*) for 5 min, a gel-like RNA pellet is formed along the tube’s side and bottom. The RNA pellet was washed with 75% ethanol, then vortexed and centrifuged at 2 to 8 °C (7500× *g*) for 5 min. The vortexing and subsequent centrifugation were repeated twice to eliminate the remaining ethanol. The RNA pellet was air-dried for about ten minutes, and then 50 μL of DNase-RNase-free water was added for RNA elution.

The quantification of isolated RNA concentration and purity was estimated by measuring optical density at 260/280 nm wavelength using a nano-drop micro-volume spectrophotometer (Thermo-Scientific, Pittsburg, PA, USA). The optimal purified density of the RNA is between 1.8 and 2.0 ng/μL. Subsequently, complementary DNA (cDNA) synthesis was performed using Revert Aid First Strand cDNA Synthesis kit by Thermo Scientific. 10 µL of RNA were mixed with 1 µL Oligo (dT)_18_ Primer and 1 µL nuclease-free water, then incubated for 5 min at 65 °C. Then, after 5 min 4 µL of 5X Reaction Buffer, 1 µL of RiboLock RNase Inhibitor, 2 µL of dNTP Mix, and 1 µL of Revert Aid Reverse Transcriptase were added to the reaction to make up a final volume of 20 µL. The final volume is incubated at 42 °C for 60 min, followed by a subsequent incubation at 70 °C for a final 5-min period.

Quantitative polymerase chain reaction (RT-PCR) was performed using 2X SYBR Green qPCR master mix (Hibrigen, Biotechnology Ltd., Gebze, Turkey). 2 µL of cDNA was mixed with 5 µL of sybr green, 1 µL of forward primer, 1 µL of reverse primer, 1.25 µL of Mgcl_2_ and 4.75 dH_2_O yielding a total of 15 μL per sample to analyze the expression patterns of rs429358 (ε4) and rs7412 (ε2). Expression analysis was conducted using the Insta Q96™ Plus Real-time PCR Detection System (HiMedia Laboratories Pvt. Ltd., Mumbai, India).

### 2.4. Statistical Analysis

The statistical analysis for this study was conducted using the SPSS software (Statistical Package for the Social Sciences 25.0, SPSS Inc., Chicago, IL, USA), a widely recognized tool for data analysis in the field. To present the findings comprehensively, qualitative data were stated in percentages, while quantitative variables were presented as the mean accompanied by the standard deviation (SD), giving an overview of the data’s central tendency and highlighting the extent of variability presented in the dataset. To thoroughly analyze gene expression, we obtained CT values and carefully compared the expression of each gene across different depots using the 2^ΔΔCT^ method. The data underwent an extensive statistical analysis, which included the comparative mean *t*-test to identify significant differences in gene expression levels between depots. Furthermore, we assessed correlations between the expression levels of the studied genes using the Spearman correlation test. A significance level of *p* < 0.05 was selected to ascertain the statistical significance of observed differences and correlations, ensuring the strength of the study’s findings.

## 3. Results

### 3.1. Population Study Characteristics

This study was conducted at NEUH (Near East University Hospital) and involved a total of 115 samples that were categorized into two groups: the patient group (62 individuals) and the control group (53 individuals). These samples were collected from the remnants of vein surgeries performed at NEUH. The patient group consisted of individuals who were confirmed to have issues in their great saphenous vein through the use of Doppler ultrasonography and blood flow assessment using physical compression. On the other hand, the control group comprised individuals who did not exhibit any abnormalities or issues in their veins. The age and gender of the study population are shown in (Table 1).

### 3.2. Gradient PCR and Gel Electrophoresis Findings

The primary objective of this research study was to thoroughly investigate and clarify the intricate expression levels of the APOE allele’s ε4 and ε2, associated with the widely recognized genetic variations rs429358 and rs7412, respectively. To analyze *APOE* gene variations, specific forward and reverse primers for the two SNPs were designed using the SnapGene system version 6.0, as seen in (Table 2). After gradient PCR products were run on the gel and visualized, the optimal annealing temperatures were determined to be 60 °C for rs429358 (ε4) and 61 °C for rs7412 (ε2) primers. Expression levels of rs429358 (ε4) and rs7412 (ε2) and the *ACTB* as a housekeeping gene were detected by qRT-PCR according to the conditions detailed in (Table 3).

### 3.3. Statistical Analysis Findings

**Figure 1:**
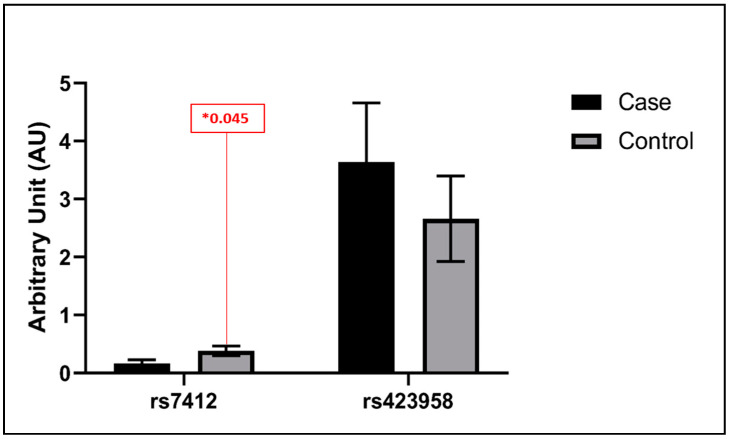
The vein tissue results for rs7412 (ε2) indicating a twofold higher expression with a significance *p*-value of 0.045 in controls compared to patients. At the same time, rs429358 (ε4) shows no significant difference in the vein tissues of both control and patient cohorts. (*) indicates a *p*-value less than 0.05, which is often considered statistically significant at a 5% significance level.

**Figure 2:**
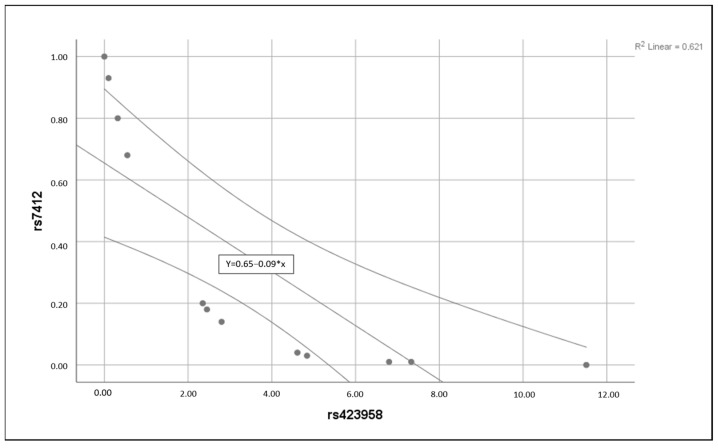
The results of the Spearman correlation test for both SNPs in vein tissues. The results unveil a statistically significant negative correlation within the vein tissue, evident from a *p*-value of (0.01).

As mentioned earlier, comparative mean *t*-test and Spearman correlation analysis were employed in our analytical approach. The results obtained from the t-test comparison were unusually enlightening, as they unveiled intriguing patterns in the vein tissue expression profiles of rs429358 (ε4) and rs7412 (ε2) in our patient and control groups. Interestingly, our investigation found that there was no apparent statistically significant difference in the expression levels of rs429358 (ε4) in the vein tissues of both control and patient cohorts. This observation indicates that the expression of this allele in vein tissue is relatively stable, highlighting its potential resistance to fluctuations related to the physiological condition of the patient. On the contrary, rs7412 (ε2) yielded a statistically significant difference in expression between the control and patient groups. The ε2 allele had two times higher expression in control compared to the patient, with a significance *p*-value of 0.045. (Figure 1). The statistical significance of this finding emphasizes the clinical implications of this genetic variation in the context of vein tissue dynamics. To broaden our analytical scope, we performed a correlation test to evaluate the association between both SNPs representing alleles within the vein tissue. The results revealed a significant negative correlation with a *p*-value of (0.01), as shown in (Figure 2). This correlation adds an additional layer of complexity to our findings, suggesting a potential association or regulatory mechanism between rs429358 (ε4) and rs7412 (ε2) within the vein tissue environment.

## 4. Discussion

Understanding the intricate genetic factors influencing susceptibility to the illness has become a focal point in cardiovascular research. Venous insufficiency is a condition characterized by impaired blood flow in the veins of the legs, resulting in inadequate return of blood to the heart and upper body [22]. The primary objective of this study was to examine the potential link between *APOE* gene variations, specifically the ε4 allele (SNP rs429358) and the ε2 allele (SNP rs7412), and the occurrence of cardiovascular disease in individuals with venous insufficiency disease.

Recent research from Taiwan found that varicose veins were associated with an increased incidence of venous thromboembolism and peripheral artery disease [7]. Additionally, data from the Framingham Heart Study demonstrated that individuals with varicose veins had a higher risk of future atherosclerotic cardiovascular disease, particularly coronary heart disease [23]. These findings suggest a possible connection between the arterial and venous vascular beds, independent of traditional cardiovascular risk factors and comorbidities. This highlights the significance of considering “venous disease” as a component of vascular disease alongside arterial vascular disease [2].

Among these genetic elements associated with cardiovascular diseases, the *APOE* gene has garnered substantial attention due to its associations with various health conditions [24]. In a case-control study investigating the signaling pathways related to structural changes in varicose veins, it was found that the expression of *APOE* genes was down-regulated in varicose veins compared to the control group [25]. In a Chinese case-control study studying the association between the *APOE* gene and lower extremity deep venous thrombosis (LEDVT), the patients had a higher frequency of the APOE E3/E4 genotype compared to healthy controls [26]. Another pilot study in the Turkish population found a relationship between ApoE3/E4 gene polymorphism and DVT [27].

Numerous studies have identified a significant association between the ε4 allele and cardiovascular diseases [28]. Additionally, it was found that the same allele was linked to a heightened susceptibility to developing hypertension [29]. A comprehensive genomic study involving a substantial sample size of 32,965 controls and 15,492 cases revealed that individuals possessing the ε4 allele exhibited an increased susceptibility to coronary heart disease (CHD) in comparison to individuals with the ε3 genetic makeup [30].

While another investigation exploring the connection between *APOE* gene polymorphism, blood lipid levels, and CAD in individuals of African Caribbean descent reported no observed correlation between *APOE* gene polymorphism and the occurrence of coronary artery disease [31], these inconsistencies may have arisen due to variations in regional and ethnic factors. In a study by Bos et al. (2021) analyzing Apolipoprotein E gene-environment interaction using data from the UK Biobank (345,659 whites without a history of coronary heart disease), carriers of the ε2 allele (rs7412) had the best overall survival rate in the stud, while the ε4 allele (rs429358) holders had the worst prognosis [12]. In the predominantly white population of Western Siberia (Russia), individuals homozygous for the ε4 allele (rs429358) exhibited a poorer survival progression after myocardial infarction. Meanwhile, the ε2 allele (rs7412) did not demonstrate any apparent protective effect when examining its association with lipid profile parameters [32].

The ε4 allele (rs429358) allele has been described as a risk factor for coronary artery diseases (CAD) and is linked to low APOE concentrations, according to a different Italian investigation [33]. On the other hand, in Greek patients with CAD, the ε4 allele (rs429358) was not related to the risk of CAD or MI, contrary to the ε2 allele (7412), which was found to be negatively associated with CAD and Ml [34]. Another study in the Greek population by Kulminski et al. in 2016, investigating the protective role of the apolipoprotein ε2 allele in age-related disease, found no higher risk for CVD or ischemic vascular events in patients with CVD carrying the ε4 allele [35]. In the Thai population, both type II diabetes and coronary artery disease (CAD) have been linked to the ε4 allele. The risk of both diseases increased twice when combined with smoking or obesity compared to controls [36].

Hypertension is widely acknowledged as the primary risk factor associated with the onset of cardiovascular and cerebrovascular diseases. Hypertension and its correlated complications have noteworthy impacts on the life expectancy of individuals affected by the condition. Additionally, they impose considerable societal and economic burdens. Significant differences can be observed in the clinical characteristics exhibited by young and middle-aged individuals diagnosed with hypertension as compared to elderly patients [37]. Numerous studies have provided evidence suggesting that the existence of the *APOE* ε4 allele (rs429358) is correlated with a heightened susceptibility to the development of hypertension. An independent study discovered that the APOE ε4 allele is an independent risk factor for recurrent intracerebral hemorrhage in hypertensive individuals of Indian descent [38]. Rao et al. (2022) study in the southern Chinese population provided evidence indicating that individuals possessing the *APOE* ε2 (rs7412) T/T genotype also exhibit a heightened susceptibility to the development of hypertension [39]. Another study by Lan et al. (2023) in the Chinese population revealed that older adults possessing the ε2 (rs7412) T/T genotype displayed an increased risk for developing hypertension, whereas this correlation was not evident among individuals in the middle-aged demographic. Furthermore, no significant association was observed between the *APOE* rs429358 polymorphisms and hypertension in both middle-aged and elderly cohorts [40].

In contrast to several preceding studies conducted by Boss et al., 2021 [12], Liu et al., 2019 [41], and Lumsden et al., 2020 [42], which reported a significant positive association between the expression of the ε4 allele of the *APOE* gene and cardiovascular diseases. The data demonstrated in our study indicated that the first SNP, rs429358, representing allele (ε4), was not significantly expressed in venous tissue between our control and patient groups. Therefore, not related to the risk of CVD, consistent with the results from (Larifla et al., 2017), which reported no association between *APOE* gene polymorphism and CAD in Afro-Caribbean people [31]. The second SNP, rs7412, which represents the APOE ε2 allele, was significantly reduced in the venous tissue of the patients. This is consistent with previous studies that have reported a decreased risk of cardiovascular disease (CVD) among carriers of the APOE ε2 allele [43,44,45]. Our findings also align with a study by Modaghegh et al. (2022), which showed the down-regulation of *APOE* gene expression in varicose veins compared to the control group [25]. Both SNPs showed a negative correlation in venous tissue. The lower expression of the rs7412 allele (ε2) in the veins of patients could indicate a lower risk of CVD in these individuals. It is important to note that the expression levels of the APOE gene can vary among individuals, and the low expression of the ε2 allele in varicose veins of patients may suggest reduced production of the associated protein. However, the specific relationship between APOE allele ε2 gene expression levels and CVD risk is complex and not yet fully understood.

## 5. Conclusions

This study examined the association between APOE alleles and cardiovascular disease (CVD) risk, focusing on the ε2 and ε4 alleles. The findings showed no significant difference in the expression of the ε4 allele between the control and patient groups. However, the expression of the ε2 allele was significantly lower in patients, suggesting a potentially lower CVD risk in individuals with venous insufficiency. The study focused on identifying SNPs in cardiovascular tissue but did not explore specific allele combinations. Limitations include selection bias in a hospital-based case-control design and limited generalizability due to the specific population from North Cyprus. Further research is needed to explore these findings and their implications.

## Figures and Tables

**Table 1:** Age and Gender of study population.

Group	Gender	Sample Size	Age
Patient	Total	*n* = 62	Mean = 57
			Median = 49
			Mode = 41
	Female	*n* = 31	Mean = 68
			Median = 52
			Mode = 66
	Male	*n* = 31	Mean = 46
			Median = 43
			Mode = 55
Control	Total	*n* = 53	Mean = 59
			Median = 63
			Mode = 64
	Female	*n* = 18	Mean = 62
			Median = 68
			Mode = 77
	Male	*n* = 35	Mean = 58
			Median = 60
			Mode = 64

**Table 2:** Primers designed for the *APOE* gene SNPs.

rs429358 T>C-ε4	Sequence	TM	Product Size
rs429358_Forward T	GCGGACATGGAGGACGTG**T**	61–62 °C	130 bp
rs429358_Reverse	GAGCCGCTTACGCAGCTTG	61 °C	
**rs7412 C>T-ε2**			
rs7412_Forward C	GCCGATGACCTGCAGAAG**C**	60–61 °C	216 bp
rs7412_Reverse	GCTGCCCATCTCCTCCATC	58–59 °C	

**Table 3:** The conditions employed for RT-qPCR APOE expression analysis.

Stage	Temperature	Time	Cycles
Initial denaturation	95 °C	2 min	1 cycle
Denaturation	95 °C	30 s	
Annealing	60–61 °C	30 s	40 cycles
Termination	72 °C	45 s	1 cycle

## Data Availability

Data are available upon request from the authors.

## Abbreviations

The following abbreviations are used in this manuscript:

CVI	Chronic venous Insufficiency
CVDs	Cardiovascular diseases
LEDVT	Lower extremity deep venous thrombosis
APOE	Apolipoprotein E
ε2	Epsilon 2
ε3	Epsilon 3
ε4	Epsilon4
SNPs	Single Nucleotide Polymorphisms
NCDs	Non-communicable diseases
*IL-6*	Interleukin-6
APOB	Apolipoprotein B
AD	Alzheimer’s disease
NEUH	Near East University Hospital
DNA	Deoxyribonucleic acid
PCR	Polymerase chain reaction
RTQ-PCR	Quantitative reverse transcription polymerase chain reaction
CT	Cycle Threshold
TM	Melting temperature
